# A Combination of Soybean and *Haematococcus* Extract Alleviates Ultraviolet B-Induced Photoaging

**DOI:** 10.3390/ijms18030682

**Published:** 2017-03-22

**Authors:** Jieun Shin, Jong-Eun Kim, Kum-Ju Pak, Jung Il Kang, Tae-Seok Kim, Sang-Yoon Lee, Ik-Hyun Yeo, Jung Han Yoon Park, Jong Hun Kim, Nam Joo Kang, Ki Won Lee

**Affiliations:** 1 Department of Agricultural Biotechnology, Seoul National University, Seoul 08826, Korea; 123456wldms@hanmail.net; 2 Research Institute of Biotechnology and Medical Converged Science, Dongguk University (Seoul), Goyang 10326, Korea; jekim14@dongguk.edu; 3 The Food and Culture Institute, Pulmuone Co., Ltd., Seoul 03722, Korea; kjparka@pulmuone.com (K.-J.P.); jikang@pulmuone.com (J.I.K.); tskim@pulmuone.com (T.-S.K.); sylee@pulmuone.com (S.-Y.L.); hyeo@pulmuone.com (I.-H.Y.); 4 Research Institute of Agriculture and Life Sciences, Seoul National University, Seoul 08826, Korea; jyoon@hallym.ac.kr (J.H.Y.P.); killrose@hotmail.com (J.H.K.); 5 School of Food Science and Biotechnology, Kyungpook National University, Daegu 41566, Korea

**Keywords:** soybean, *Haematococcus*, ultraviolet B, photoaging

## Abstract

Soybean-derived isoflavones have been investigated for their preventative effects against UV-induced symptoms of skin damage including wrinkle formation and inflammation. *Haematococcus pluvialis* is a freshwater species of Chlorophyta that contains high concentrations of the natural carotenoid pigment astaxanthin. Astaxanthin is known to be involved in retinoic acid receptor (RAR) signaling and previously been associated with the inhibition of activator protein (AP)-1 dependent transcription. Based on previous studies, we hypothesized that a combination of soy extract (SE) and *Haematococcus* extract (HE) may prevent UVB-induced photoaging through specific signaling pathways, as measured by UVB-induced wrinkling on hairless mice skin and expression changes in human dermal fibroblasts (HDFs). The 1:2 ratio of SE and HE mixture (SHM) showed the optimal benefit in vivo. SHM was found to inhibit wrinkle formation via the downregulation of matrix metalloproteinase (MMP)-1 mRNA and protein expression. SHM also inhibited mitogen-activated protein kinase (MAPK) phosphorylation and the transactivation of AP-1 which plays an important role in regulating MMP expression. These results highlight the potential for SHM to be developed as a therapeutic agent to prevent UVB-induced skin wrinkling.

## 1. Introduction

As life expectancy in a society increases, more individuals choose to invest in improving their appearance and reverse the signs of aging [1]. The skin is the most conspicuous measure of aging, and it is thus important to many to maintain skin health [2]. Skin is the largest organ of the human body and plays a crucial role as a protective barrier against the external environment [3]. A complex interaction between intrinsic and extrinsic factors results in the phenomenon of aging [4], with intrinsic mechanisms proceeding naturally through senescence characterized by dryness and fine wrinkling of the skin [5]. In parallel, extrinsic skin aging occurs due to environmental factors such as smoking, ultraviolet (UV) light exposure [6] and air pollution [7]. Extrinsic aging caused by UV exposure from sunlight (known as photoaging) contributes to an estimated ~90% of the overall skin changes [8]. UV light can also cause direct damage to DNA leading, to the upregulation of pro-inflammatory cytokines such as nuclear factor kappa B (NF-κB) [9]. Clinically, the photoaged phenotype includes sagging and deeply wrinkled skin [10].

The sunlight spectrum is divided into ultraviolet (UV), infra-red (IR), and visible light according to wavelength. UV comprises UVA (320–400 nm), UVB (280–320 nm), and UVC (200–280 nm). All UVC is blocked by the ozone layer, while UVA and UVB penetrate to the surface of the Earth and accelerate skin aging [11]. UVB contains more energy than UVA, and can cause sunburn, tanning, immune suppression, photoaging, and photo-carcinogenesis [12]. Chronic exposure to UV changes the composition of the dense collagen-rich extracellular matrix (ECM). ECM is composed of connective tissue and basement membrane proteins such as elastin, glycosaminoglycans and interstitial collagen [13]. In human skin, UV radiation can elevate the levels of various matrix metalloproteinases (MMPs) including MMP-1, MMP-3, and MMP-9 [14,15]. Of these, MMP-1, fibroblast collagenase, is the key enzyme responsible for breaking down dermal components in the ECM [16,17]. Once the elevated levels of MMP-1 initiate degradation of fibrillary type I and III collagen, further processing is followed by MMP-3 and MMP-9 [18]. Therefore, MMP-1 plays an important role in the initiation of UVB-induced wrinkle formation through ECM degradation. UV-induced MMP-1 overexpression is followed by the upregulation of the mitogen-activated protein kinase (MAPK) signaling pathway through various factors such as cytokines and growth factor receptors [15]. Activator protein (AP)-1 is a transcription factor and a major effector of the MAPK pathway in regulating MMPs expression. AP-1 forms heterodimer complexes between Jun, Fos or activating transcription factor proteins [19]. Because chronic exposure to UVB can promote photoaging, the inhibition of MMP-1 expression may result in preventative effects.

Soy foods have been consistently consumed in Asian countries for both nutritional and medical reasons [20]. Soybean is a high-quality protein source that contains several key bioactive compounds [21]. Among other components, isoflavones are associated with beneficiary effects on human health, with anti-carcinogenic properties and estrogen-like effects based on their diphenolic structure [22,23]. In our previous study, we investigated the effects of soy-derived isoflavonoids UV-induced skin damage and the specific molecular targets involved. Coumestrol is a soybean isoflavonoid that potently counteracts UVB-induced skin wrinkle formation via regulation of the Ras/MEK/ERK and Akt/p70S6K pathways through targeting FLT3 kinase [24]. Biochanin A, another isoflavone found in soy, inhibits solar UV-induced cyclooxygenase (COX)-2 expression by directly targeting MLK3 kinase, resulting in downstream effects on the MKK4/JNK/c-Jun and MKK3/6/p38/MSK signaling pathways [25]. Furthermore, soybean isoflavones, especially in the aglycone form such as genistein and daidzein, are considered to have beneficial effects on the skin through the stimulation of fibroblast proliferation, reduction of collagen degradation and various other mechanisms [26]. Based on the previous studies, we hypothesized that soy isoflavones can effectively improve UVB-induced photoaging through specific molecular mechanisms.

*H. pluvialis*, a green algae, is the richest known biological source of natural astaxanthin. Astaxanthin is a carotenoid pigment present in salmon, shrimp, lobster, and fish eggs [27]. Astaxanthin accumulation in *H. pluvialis* can be observed only in encysted cells as a defense mechanism when they are under unfavorable growth conditions such as elevated temperature, nitrogen or phosphate limitation, abnormal pH or high salinity [28,29]. Animals cannot synthesize the astaxanthin, and the only source is from the diet [27]. Carotenoids exhibit biological activity after being conversion to retinoids [30]. Most carotenoids such as β-carotene (which can be converted into vitamin A) have numerous beneficial effects on human health through direct binding with the retinoic acid receptor (RAR) [31]. Astxanthin is a non-provitamin A carotenoid and is not thought to undergo retinol conversion [27], however, some studies suggested that astaxanthin could be involved in RAR or retinoid signaling [30]. In a previous study, non-provitamin A carotenoids including astaxanthin were shown to stimulate RARs such as retinoic acid, resulting in the production of hyaluronan, an important component of the ECM [32]. It remains to be determined whether astaxanthin could be converted into β-carotene or retinol in vivo [33]. RARα, -β, and -γ forms are all activated by retinoids and are associated with the inhibition of AP-1-dependent transcription. RARα prevents Jun/AP-1 from binding to DNA, leading to direct inhibition of the interaction between RARs and c-Jun [34]. However, the mechanism by which non-provitamin A exhibits such diverse biological activities remains to be fully elucidated.

Although soybean extract (SE) and *Haematococcus* extract (HE) have been extensively investigated, the anti-wrinkling effects of a combined SE and HE mixture have not been reported.

## 2. Results

### 2.1. Oral Administration of SHM Reduces UVB-Induced Skin Wrinkling in Hairless Mice

**Figure 1:**
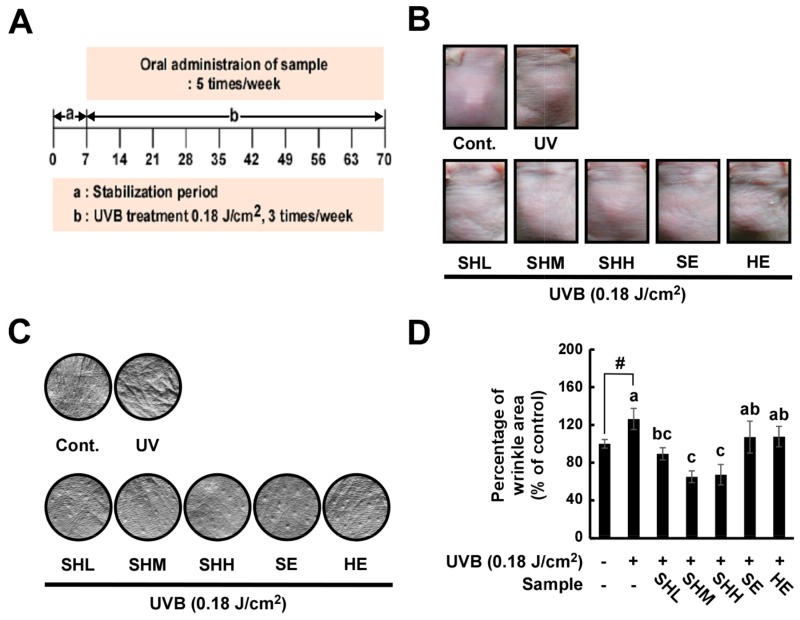

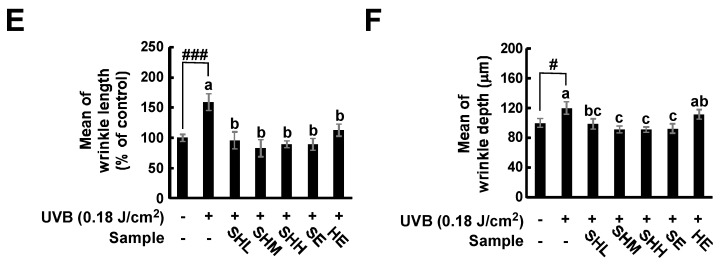
Inhibitory effects of soybean and *Haematococcus* extract mixture on UVB-induced wrinkle formation in hairless mice: (**A**) schematic diagram of the experiment, each group consisted of 6–8 mice; (**B**,**C**) dorsal skins of hairless mice were exposed to UVB for nine weeks; and (**D**–**F**) percentage of skin classified as wrinkled (**D**); mean of winkle length (**E**); and depth (**F**) were analyzed by Skin-Visiometer software after nine weeks of UVB treatment. Data represent the means ± SEM (*n* = 6–8). The vehicle was 0.5% sodium carboxymethylcellulose. Means with letters # (*p* < 0.05) and ### (*p* < 0.001) within a graph are significantly different between untreated control and UVB treated group. Means with letters a–c within a graph are significantly different from each other at *p* < 0.05. The doses of sample and mixture ratio were as follows: SE (11.67 mg/kg of SE), HE (53.33 mg/kg of HE), SHL (11.67 mg/kg of SE and 13.33 mg/kg of HE, about 1:1 ratio of SE and HE), SHM (11.67 mg/kg of SE and 26.67 mg/kg of HE, about 1:2 ratio of SE and HE) and SHH (11.67 mg/kg of SE and 53.33 mg/kg of HE, about 1:4 ratio of SE and HE).

Hairless mice were exposed to UVB on their dorsal skins for nine weeks (Figure 1A). The area of wrinkling, length and depth of the wrinkles were analyzed using skin replicas. The doses of sample and mixture ratio were as follows: SE (11.67 mg/kg of SE), HE (53.33 mg/kg of HE), SHL (11.67 mg/kg of SE and 13.33 mg/kg of HE, about 1:1 ratio of SE and HE), SHM (11.67 mg/kg of SE and 26.67 mg/kg of HE, about 1:2 ratio of SE and HE) and SHH (11.67 mg/kg of SE and 53.33 mg/kg of HE, about 1:4 ratio of SE and HE). UVB-induced wrinkling was found to be reduced in the groups receiving administration of the SE and HE mixture (Figure 1B,C). A greater decrease in wrinkling was observed in the SHM and SHH group compared to the animals receiving only SE or HE (Figure 1D). All treated groups exhibited reduced wrinkle lengths compared to the UVB-irradiated group (Figure 1E), and similar results were found for wrinkle depth, except for HE (Figure 1F).

### 2.2. SHM Prevents UVB-Induced Increases in Epidermal Thickness and Collagen Degradation in Hairless Mice

**Figure 2:**
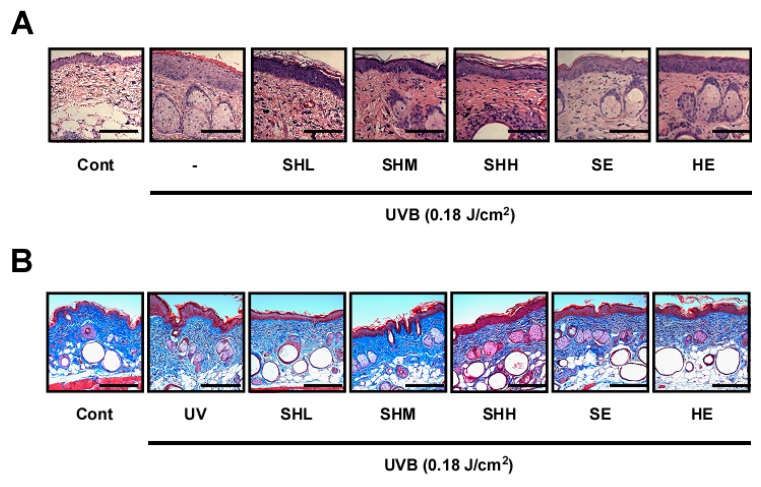

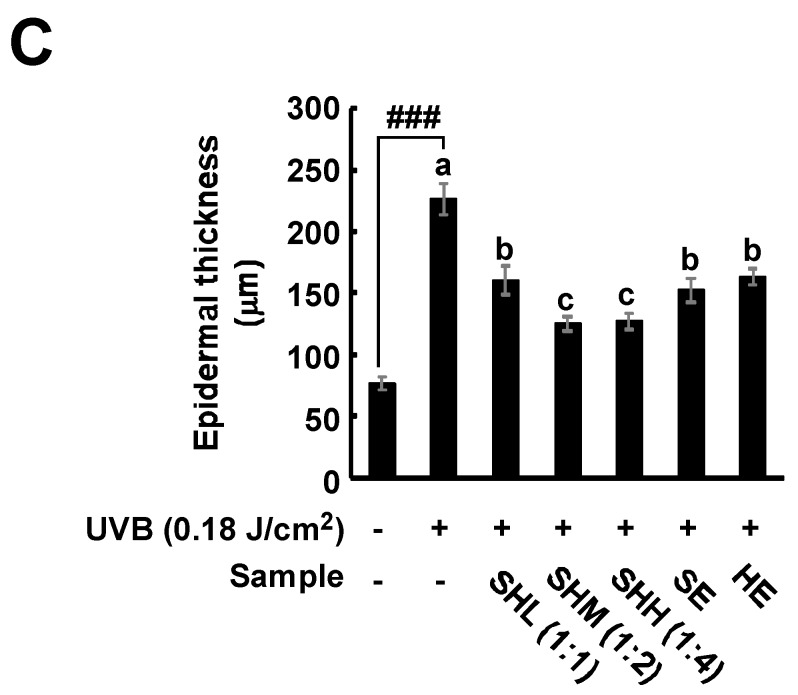
Effect of SHM on UVB-induced skin inflammation and collagen degradation in hairless mice: (**A**,**B**) Dorsal skin sections were stained with hematoxylin and eosin (H&E). Scale bar is 200 μm. The epidermal thicknesses were quantified using Image J software analysis as described in the Materials and Methods. Means with letters a–c within a graph are significantly different from each other at *p* < 0.05. Means with letters ### within a graph are significantly different between untreated control and UVB treated group at *p* < 0.001. Data represent the means ± SEM (*n* = 5); (**C**) Masson’s trichrome staining for the visualization of collagen fibers as described in the Materials and Methods. Collagen fibers appear blue.

When human skin is acutely exposed to UVB, epidermal hyperplasia [35] and collagen degradation occurs [36]. To determine the effect of UVB exposure on epidermal thickness and collagen degradation, mouse skin samples were stained with hematoxylin and eosin and Masson’s trichrome. Epidermal thickness was reduced in all treatment groups (Figure 2A). A greater decrease in epidermal thickness was observed for the SHM group compared to the animals that received only SE or HE (Figure 2C). Collagen fiber levels were also recovered in the treatment groups (Figure 2B).

### 2.3. SHM Suppresses UVB-Induced MMP-1 Overexpression in Cultured Primary Human Dermal Fibroblasts

**Figure 3:**
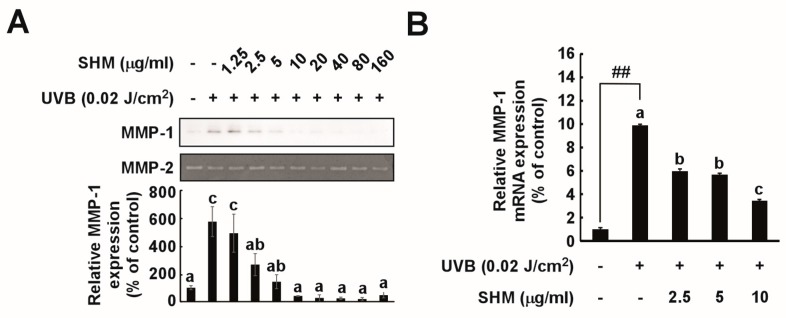

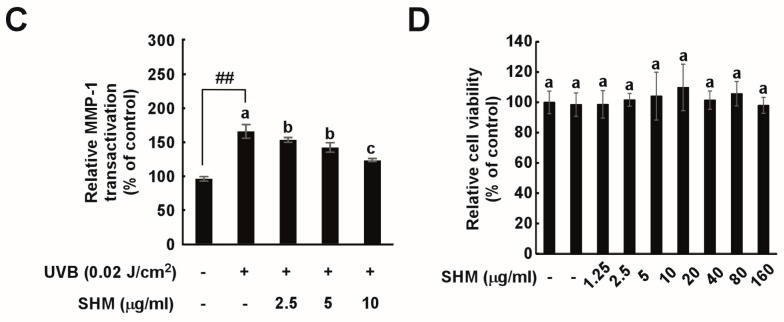
Effect of SHM on UVB-induced MMP-1 transcription in cultured primary human dermal fibroblasts: (**A**) Expression of MMP-1 was determined by Western blot. MMP-2 was used as a loading control. Cells were pretreated with SHM at the indicated concentrations for 1 h, and then further treated with 0.02 J/cm^2^ UVB for 48 h at 37 °C. MMP-1 expression data were quantified using Image J software analysis. Data (*n* = 3) represent the means ± SD; (**B**) MMP-1 mRNA levels for the SHM group were analyzed by real-time quantitative PCR. Cells were pretreated with SHM at the indicated concentrations for 1 h, and then further treated with 0.02 J/cm^2^ UVB for 48 h at 37 °C. Data (*n* = 3) represent the means ± SD; (**C**) MMP-1 transactivation by SHM, measured using a luciferase reporter gene assay as described in the Materials and Methods. Cells were pretreated with SHM at the indicated concentrations for 1 h, and then further treated with 0.02 J/cm^2^ UVB for 24 h at 37 °C. Data (*n* = 3) represent the means ± SD; (**D**) Cell viability after SHM treatment. Viability was measured using an MTT assay as described in the Materials and Methods. Cells were pretreated with SHM at the indicated concentrations for 48 h at 37 °C. Data (*n* = 4) represent the means ± SD. Means with letters a–c within a graph are significantly different from each other at *p* < 0.05. Means with letters ## within a graph are significantly different between untreated control and UVB treated group at *p* < 0.05.

To elucidate the molecular mechanisms underlying the anti-wrinkling effects of SHM, an in vitro study was performed. SHM was observed to reduce MMP-1 protein expression in a concentration-dependent manner (Figure 3A), and effectively suppressed UVB-induced MMP-1 mRNA level and transactivation (Figure 3B,C). These inhibitory effects of SHM were apparent within a concentration range that did not affect cell viability (Figure 3D).

### 2.4. SHM Significantly Downregulates UVB-Induced AP-1 Transactivation and Modulates UVB-Induced Signal Transduction in Cultured Primary Human Dermal Fibroblasts Independently of Akt and TIMP-1

**Figure 4:**
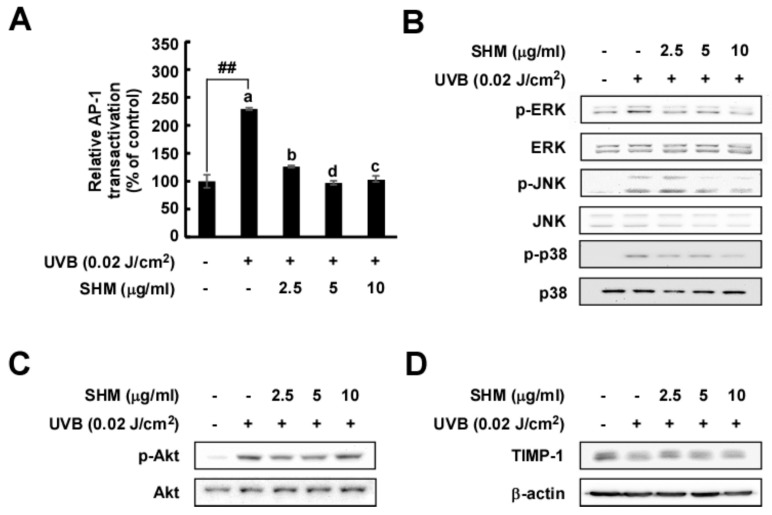
Effect of SHM on UVB-induced AP-1 protein expression and signaling pathways in cultured primary HDF and TIMP-1 cell lines. (**A**) AP-1 transactivation by SHM was measured using a luciferase reporter gene assay as described in the Materials and Methods. Cells were pretreated with SHM at the indicated concentrations for 1 h, and then further treated with 0.02 J/cm^2^ UVB for 36 h at 37 °C. Data (*n* = 3) represent the means ± SD; Means with letters ## within a graph are significantly different between untreated control and UVB treated group at *p* < 0.05; (**B**–**D**) Phosphorylated and total protein levels were conducted by Western blot using specific antibodies. Cells were pretreated with SHM at the indicated concentrations for 1 h, and then further treated with 0.02 J/cm^2^ UVB for 30 min at 37 °C.

MMP-1 transcription is regulated by activator protein (AP)-1, a transcription factor activated by UVB irradiation [37]. We observed that SHM effectively suppressed UVB-induced AP-1 transactivation (Figure 4A). Based on previous findings, the MAPK signaling pathway plays a crucial role in regulating MMP-1 expression [37]. To identify the possible target responsible for reducing MMP-1 expression, we evaluated MAPK signal transduction. SHM attenuated UVB-induced phosphorylation of major MAPK family members via AP-1 transactivation (Figure 4B). In contrast, SHM had no significant inhibitory effect on Akt signal transduction (Figure 4C). TIMP-1 is a natural inhibitor of MMPs, which are involved in degradation of the extracellular matrix [25]. SHM had no inhibitory effect on TIMP-1 protein expression (Figure 4D).

## 3. Discussion

Skin aging can be accelerated by chronic exposure to sunlight in a process called photoaging [38,39]. Within the solar UV spectrum, UVB (280–320 nm) can accelerate various physiological changes in human cells. Chronic UVB exposure induces wrinkle formation, pigmentary changes, and increases in laxity [40]. In epidermal and dermal cells, AP-1 forms complexes to regulate the transcription of MMPs [41]. The MMPs are members of an enzyme family that require a zinc ion within their active site for catalytic activity [42]. The active MMPs are generated by proteolytic cleavage of an inactive form referred to as a zymogen. TIMPs are inhibitors of MMPs, and regulate this activation process [3]. MMPs in turn contribute to the balanced and regulated degradation of ECM proteins [41], and play a pivotal role in UVB-induced wrinkling by the breakdown of collagen in the dermis. MMP-1 cleaves α-chains of native triple helical type I and III collagens after glycine (Gly) in a specific sequence present in collagen molecules [2,43]. Therefore, the inhibition of UVB-induced MMP-1 overexpression may be a promising strategy to prevent photoaging.

Albino hairless mice (Skh-1) are a popular mouse model for the study of chronic UVB exposure. Chronically UVB-exposed Skh-1 mice can develop wrinkles, which appear as prominent horizontal creases on the dorsal skin [44]. Further verification of the effects observed in this study is needed in clinical settings.

To identify the underlying mechanisms involved in UVB-induced wrinkle prevention by SHM treatment, an in vitro study was performed. Chronic exposure of UVB induced MMP-1 overexpression, leading to the breakdown of collagen. In the wrinkle formation process, the ECM containing connective tissue and basement membrane protein play critical roles. Dermal fibroblasts are known to produce and secrete MMP-1, which plays a key role in dermal remodeling [45]. Therefore, HDFs were used to investigate the mechanisms associated with the anti-wrinkle effect of SHM.

For physiological relevance, Skh-1 hairless mice in this study were exposed to UVB at a dose of 180 mJ/cm^2^ and HDFs were exposed to UVB at a dose of 0.002 J/cm^2^. These UVB doses represent reasonable exposure to the sun for approximately two hours in mid-April in New York City [46].

We found that the oral administration of SHM reduced UVB-induced wrinkle formation and prevented UVB-induced collagen degradation in the hairless mice. It was additionally observed that SHM exhibits an anti-wrinkle effect by suppressing UVB-induced MMP-1 protein expression and MMP-1 gene transcription in HDFs. The MMP-1 suppression was associated with a reduction in UVB-induced AP-1 expression, a major transcription factor of MMP-1, activity and the MAPK signaling pathway. Further studies are ongoing to determine the precise molecular target responsible.

## 4. Materials and Methods

### 4.1. Chemicals and Reagents

Dulbecco’s modified eagle medium (DMEM) was purchased from Welgene (Gyeongsan, Korea). Fetal bovine serum (FBS) was purchased from Sigma-Aldrich (St. Louis, MO, USA). The MMP-1 antibody was obtained from R&D systems Inc. (Minneapolis, MN, USA). Antibodies against total c-Jun N-terminal kinase 1/2 (JNK1/2), total p38 and phosphorylated extracellular-signal regulated kinase 1/2 (ERK1/2) (Thr202/Tyr204), total ERK1/2 were purchased from Santa Cruz Biotechnology (Santa Cruz, CA, USA). Antibodies against phosphorylated JNK1/2 (Thr183/Tyr185), phosphorylated-p38 (Thr180/Tyr182), phosphorylated p90RSK (Thyr359/Ser363), total p90RSK and a tissue inhibitor of metalloproteinases (TIMP)-1 were purchased from Cell Signaling (Danvers, MA, USA). 3-[4,5-dimethylatiazol-2-yl]-2,5 diphenyltetrazolium bromide (MTT) powder was purchased from USB Co. (Cleveland, OH, USA). Penicillin-Streptomycin solution was purchased from Mediatech, Inc. (Manassas, VA, USA). Protein assay reagent kits were obtained from Bio-Rad Laboratories (Hercules, CA, USA).

### 4.2. Sample Preparation

SE was provided by Skyherb Co., Ltd. (Seoul, Korea). Black soybean, *Glycine max* (L.) Merr, was converted to soybean meal using a vibrating screen cold press. The soybean meal was then extracted with ethanol and concentrated under reduced pressure. SE was extracted after purification, elution, and spray drying. The total isoflavone content in SE was 40.49% and the contents of genistein, daidzein, genistin, and daidzin were 19.75%, 19.52%, 0.15%, and 0.83% respectively. The SE was dissolved in 0.5% sodium carboxymethylcellulose for animal treatment and in dimethylsulfoxide for cell treatment. HE was provided by Fuji Chemical Industry Co. (Toyama, Japan). Microalgae HE (AstaREAL^®^L10, Gustavsberg, Sweden) and all other component were mixed with a high-shear mixer. AstaREAL^®^EL25 was obtained through encapsulation, granulation, and a sieving process. The total astaxanthin content was 2.5%–2.7% representing 2.6%–2.9% of the total carotenoids in AstaREAL^®^EL25. HE was dissolved in 0.5% sodium carboxymethylcellulose for animal treatment and in dimethylsulfoxide for cell treatment.

### 4.3. Animals and Treatments

Five-week-old female albino hairless mice (Skh-1) were provided by Orient Bio (Seongnam, Korea). Animals were acclimated for 1 week prior to the study and had free access to water and food. The Institutional Animal Care and Use Committee (No. 2014-0057, 19 May 2014) of the Biomedical Research Institute, Kyongpook National University approved all experimental protocols. Six to eight mice were allocated into seven groups.

The test compounds and vehicle (0.5% sodium carboxymethylcellulose) were orally administered for nine weeks. Body weight and food intake were monitored on a weekly basis. A UVB-induced photoaging procedure was undertaken, as described previously. A UVB irradiation device that included a TL20W/12RS UV lamp (Philips, Eindhoven, The Netherlands) with an emission spectrum between 275 and 380 nm (peak, 310–315 nm) served as the UV source. Initially, the minimal UVB dose on the dorsal skin of mice as was measured as the minimal edema dose (MEdD) comparable with the minimal erythema dose in human skin. In contrast to human skin, the mouse skin showed peak responses to UVB primarily as edema, an increased thickness of the dorsal skin at 48 h post-UVB irradiation. The irradiation dose was increased weekly by 0.5 MEdD (1 MEdD = 100 mJ/cm^2^) up to 2 MEdD and then maintained at 2 MEdD. UVB irradiation was stopped after nine weeks (Figure 1A).

### 4.4. Cell Culture and Treatments

Primary HDFs were isolated from the outgrowth of foreskin obtained from 12 year-old healthy volunteers from Chung JH Laboratory (Seoul National University Hospital, Seoul, Korea) with the approval of the Institutional Review Board at Seoul National University Hospital (No. H-1101-116-353) and Seoul National University (No. E1408/001-002). HDFs were cultured in DMEM with 10% (*v*/*v*) FBS and 1% (*v*/*v*) penicillin/streptomycin at 37 °C and 5% CO_2_. UVB irradiation was performed on serum-starved monolayer cultures. HDFs were treated with SHM, which was dissolved in 50% ethanol, and exposed to UVB at a dose of 0.02 J/cm^2^ using the UVB source (Bio-Link Crosslinker, Vilber Lourmat, Marne-la-Vallée CEDEX 3, France) set at a spectral peak of 312 nm.

### 4.5. Cell Viability

Cell cytotoxicity was measured by MTT assay. HDFs were cultured in 96-well plates at a density of 2 × 10^3^ cells/well and incubated in DMEM-10% FBS containing penicillin/streptomycin at 37 °C in a 5% CO_2_ atmosphere. Cells were starved in serum-free DMEM for 24 h. The cells and each sample were incubated for 22 h at 37 °C, followed by treatment with MTT solution for 2 h. The medium was removed and formazan crystals were dissolved by the addition of dimethyl sulfoxide (DMSO). The absorbance at 570 nm was then measured using a microplate reader (Molecular Devices, Sunnyvale, CA, USA) and the SHM-treated and non-treated cells were compared.

### 4.6. Determination of Wrinkle Formation

To determine the severity of wrinkling, each hairless mouse was anesthetized and the UVB-exposed dorsal skin (wrinkle formation area) was photographed. Skin wrinkle replicas were made with silicon rubber (Silflo Dental Impression Materials, Potters Bar, UK) from the backs of unstrained mice. The skin replicas were photographed using a coupling charge system video camera. Using Skin-Visiometer SV 600 software (CK Electronic GmbH, Köln, Germany), we analyzed skin wrinkle. The visiometer is a computerized instrument that creates a skin microrelief map from the replica using a light transmission method.

### 4.7. Hematoxylin and Eosin Staining

To evaluate epidermal thickness, hematoxylin and eosin staining was performed. Mouse skin samples were fixed with 10% neutral-buffered formalin, and embedded in paraffin. Serial sections (4 µm) were mounted onto slides. After deparaffinizing, skin sections were re-hydrated and stained with hematoxylin solution for 5 min, before the slides were washed and stained in counterstain in eosin Y solution for 30 s. Next, the slides were dehydrated through 95% alcohol and washed in absolute alcohol for 5 min each. Lastly, the slides were incubated in xylene overnight to remaining water. Skin sections were examined at 400× magnification using an Olympus AX70 light microscope (Tokyo, Japan).

### 4.8. Masson’s Trichrome Staining

To evaluate collagen in the dermis, Masson’s trichrome staining was performed. Mouse skin samples were fixed, embedded, and deparaffinized as described above. After deparaffinizing, we stained skin sections with hematoxylin for 5 min. The slides were then washed and stained in biebrich scarlet and acid fuchsin. Then, the slides were put in phosphomolybdic-phosphotungstic acid for 10 min and aniline blue for 5 min to stain the collagen. The slides incubated in 1% acetic acid for 15 min, before dehydration. Skin sections were examined at 400× magnification using an Olympus AX70 light microscope (Tokyo, Japan).

### 4.9. Western Blot and Zymography

Primary HDFs were cultured for 48 h, and the cells were incubated in serum-free DMEM for 24 h, before treatment with various concentrations of SHM (2.5, 5, 10 µg/mL) for 1 h, followed by UVB (0.02 J/cm^2^) irradiation. The media was harvested on ice, and then centrifuged at 18,620× *g* for 10 min. The protein concentration was measured using a protein assay reagent kit as described by the manufacturer. The cells were lysed with lysis buffer (10 mM Tris (pH 7.5), 150 mM NaCl, 1% Triton X-100, 5 mM EDTA, 1 mM DTT, 0.1 mM phenylmethylsulfonyl fluoride (PMSF), 10% glycerol and protease inhibitor cocktail tablet) on ice for 30 min, prepared and centrifuged at 18,000× *g* for 15 min. The proteins were separated electrophoretically using a 10% SDS gel and transferred onto a nitrocellurose membrane (EMD Millipore, Billerica, MA, USA). The membrane was blocked in 10% bovine serum albumin for 1 h, and then incubated with primary antibody at 4 °C over 4 h. Protein bands were detected using a chemiluminescence detection kit (GE Healthcare, London, UK) after hybridization with the HRP-conjugated secondary antibody (Life Technologies, Waltham, MA, USA).

Zymography was used to determine the activity of secreted MMP-2 and was performed in 12% native gels in gelatin (0.1% *w*/*v*). The protein samples were mixed with loading buffer (0.1% bromophenol blue, 10% SDS, 25% glycerol and 0.25 M Tris (pH 6.8)), and then run on 12% SDS-PAGE gels without denaturation. Afterward, the gels were washed with renaturating buffer (Life Technologies) for 30 min and incubated for 24 h at 37 °C in developing buffer (Life Technologies). After reaction, we stained the gels using 0.5% Coomassie brilliant blue in 10% acetic acid.

### 4.10. Real-Time Quantitative PCR

Primary HDFs were treated with SHM for 24 h and harvested in RNAiso Plus (Takara Bio Inc., Shiga, Japan). RNA was quantified using a NanoDrop ND-2000 spectrophotometer (Thermo Fisher Scientific, Waltham, MA, USA). After RT with oligo-dT primers using a PrimeScriptTM 1st strand cDNA synthesis kit (Takara Bio Inc.), RT-PCR was performed using IQ SYBR (Bio-Rad Laboratories) and 2 µL of cDNA in triplicate with glyceraldehyde 3-phosphate dehydrogenase (GAPDH) as an internal control. Amplification consisted of 44 cycles at 95 °C for 10 s, 60 °C for 30 s, and 72 °C for 30 s. PCR was performed with a CFX Connect™ Real-Time PCR Detection System (Bio-Rad Laboratories, Hercules, CA, USA). cDNA was probed with the following primers: MMP-1 forward (5′-ATT CTA CTG ATA TCG GGG CTT TGA-3′); MMP-1 reverse (5′-ATG TCC TTG GGG TAT CCG TGT AG-3′); GAPDH forward (5′-ATT GTT GCC ATC AAT GAC CC-3′); GAPDH reverse (5′-AGT AGA GGC AGG GAT GAT GT-3′).

### 4.11. Luciferase Reporter Gene Assay

The lentiviral expression vectors, including pGF-AP1-mCMV-EF1-Puro (System Biosciences, Palo Alto, CA, USA), and packaging vectors, including psPAX and pMD2.0G, were purchased from Addgene Inc (Cambridge, MA, USA). pGF-MMP-1-mCMV-EF1-puro vector was generously provided by Dr, Sung-Keun Jung (Korea Food Research Institute, Seongnam, Korea). The MMP-1 promoter was cloned into the pGF vector [47]. pGF-AP1-mCMV-EF1-Puro vectors and the packaging vectors (psPAX and pMD2.0G) were then transfected into HEK293T cells using jetPEI following the manufacturer’s instructions. The viral particles were prepared by filtration using a syringe filter (0.45 mm), then combined with 8 µg/mL polybrenes (EMD Millipore, Billerica, MA, USA) and infected into 60% confluent HDFs for 24 h. The cell culture medium was changed with fresh medium for 24 h before the cells were selected for using puromycin (Sigma-Aldrich) over 36 h. HDFs were cultured for 48 h and then starved in serum-free DMEM for 24 h. After starvation, HDFs were treated with various concentrations of SHM for 1 h, followed by 0.02 J/cm^2^ UVB irradiation. Cell extracts were prepared with reporter lysis buffer (Promega, Medison, WI, USA). HDFs for MMP-1 and AP-1 were lysed after 36 h and the extracts used in the luciferase assay. MMP-1 and AP-1 activity in HDFs was determined using a luciferase assay kit buffer (Promega, Medison, WI, USA), as described by the manufacturers.

### 4.12. Statistical Analysis

Statistical analyses were performed using one-way ANOVA followed by Duncan’s multiple range test, and *p*-values of less than 0.05 were considered statistically significant.

## 5. Conclusions

Oral administration of SHM prevents UVB-induced skin wrinkling in hairless mice. SHM has an inhibitory effect on wrinkle formation through MMP-1 regulation which results from the inhibition of AP-1 activity. SHM markedly suppresses the MAPK signaling pathway, although how this precisely occurs is yet to be determined. SHM therefore has potential for development as a therapeutic agent for the prevention of UVB-induced wrinkling.

## Abbreviations

NF-κB	Nuclear Factor kappa B
MMP	Directory of open access journals
GAPDH	Glyceraldehyde 3-Phosphate Dehydrogenase
MAPK	Mitogen-Activated Protein Kinase
COX	Cyclooxygenase
SE	Soybean extract
HE	Haematococcus extract
AP-1	Activator Protein
UV	Ultraviolet
